# Early detection of pancreatic cancer by comprehensive serum miRNA sequencing with automated machine learning

**DOI:** 10.1038/s41416-024-02794-5

**Published:** 2024-08-28

**Authors:** Munenori Kawai, Akihisa Fukuda, Ryo Otomo, Shunsuke Obata, Kosuke Minaga, Masanori Asada, Atsushi Umemura, Yoshito Uenoyama, Nobuhiro Hieda, Toshihiro Morita, Ryuki Minami, Saiko Marui, Yuki Yamauchi, Yoshitaka Nakai, Yutaka Takada, Kozo Ikuta, Takuto Yoshioka, Kenta Mizukoshi, Kosuke Iwane, Go Yamakawa, Mio Namikawa, Makoto Sono, Munemasa Nagao, Takahisa Maruno, Yuki Nakanishi, Mitsuharu Hirai, Naoki Kanda, Seiji Shio, Toshinao Itani, Shigehiko Fujii, Toshiyuki Kimura, Kazuyoshi Matsumura, Masaya Ohana, Shujiro Yazumi, Chiharu Kawanami, Yukitaka Yamashita, Hiroyuki Marusawa, Tomohiro Watanabe, Yoshito Ito, Masatoshi Kudo, Hiroshi Seno

**Affiliations:** 1 Department of Gastroenterology and Hepatology, Kyoto University Graduate School of Medicine, 54 Shogoin-Kawahara-cho, Sakyo-ku, Kyoto Japan; 2 Research and Development Division, ARKRAY, Inc., Yousuien-nai, 59 Gansuin-cho, Kamigyo-ku, Kyoto Japan; 3 Department of Gastroenterology and Hepatology, Kindai University Faculty of Medicine, Osaka, Japan; 4 Department of Gastroenterology and Hepatology, Osaka Red Cross Hospital, Osaka, Japan; 5 Department of Pharmacology, Kyoto Prefectural University of Medicine, Kyoto, Japan; 6 Department of Gastroenterology and Hepatology, Japanese Red Cross Wakayama Medical Center, Wakayama, Japan; 7 Department of Gastroenterology, Otsu Red Cross Hospital, Shiga, Japan; 8 Department of Gastroenterology and Hepatology, Kitano Hospital, Tazuke Kofukai Medical Research Institute, Osaka, Japan; 9 Department of Gastroenterology, Tenri Hospital, Nara, Japan; 10 Department of Gastroenterology and Hepatology, Shiga General Hospital, Shiga, Japan; 11 Department of Gastroenterology, Hyogo Prefectural Amagasaki General Medical Center, Amagasaki, Japan; 12 Department of Gastroenterology and Hepatology, Kyoto Katsura Hospital, Kyoto, Japan; 13 Department of Gastroenterology and Hepatology, Kobe City Nishi-Kobe Medical Center, Kobe, Japan; 14 Division of Gastroenterology, Shinko Hospital, Kobe, Japan; 15 Department of Gastroenterology and Hepatology, Takatsuki Red Cross Hospital, Takatsuki, Japan; 16 Department of Gastroenterology and Hepatology, Kyoto Prefectural University of Medicine, Kyoto, Japan

**Keywords:** Cancer screening, Pancreatic cancer

## Abstract

**Background:**

Pancreatic cancer is often diagnosed at advanced stages, and early-stage diagnosis of pancreatic cancer is difficult because of nonspecific symptoms and lack of available biomarkers.

**Methods:**

We performed comprehensive serum miRNA sequencing of 212 pancreatic cancer patient samples from 14 hospitals and 213 non-cancerous healthy control samples. We randomly classified the pancreatic cancer and control samples into two cohorts: a training cohort (*N* = 185) and a validation cohort (*N* = 240). We created ensemble models that combined automated machine learning with 100 highly expressed miRNAs and their combination with CA19-9 and validated the performance of the models in the independent validation cohort.

**Results:**

The diagnostic model with the combination of the 100 highly expressed miRNAs and CA19-9 could discriminate pancreatic cancer from non-cancer healthy control with high accuracy (area under the curve (AUC), 0.99; sensitivity, 90%; specificity, 98%). We validated high diagnostic accuracy in an independent asymptomatic early-stage (stage 0-I) pancreatic cancer cohort (AUC:0.97; sensitivity, 67%; specificity, 98%).

**Conclusions:**

We demonstrate that the 100 highly expressed miRNAs and their combination with CA19-9 could be biomarkers for the specific and early detection of pancreatic cancer.

## Background

Pancreatic cancer is the 4th leading cause of cancer-related deaths worldwide and is projected to become the second most lethal tumor by 2030 [1]. One of the cancers with the poorest prognosis, pancreatic cancer has a 5-year survival rate of 12% [2]. At the time of diagnosis, <20% of patients present with localized [3] and potentially resectable and curative disease, 30–35% have locally advanced tumors, and the remaining 50–55% of patients present with metastatic disease [4]. Five-year survival rates of patients with stage 0 (Union for International Cancer Control staging), IA, and IB pancreatic cancer were 85.8%, 68.7%, and 59.7%, respectively [5]. If tumor cells invade the surrounding tissues and spread to distant organs, the 5-year survival rate decreases to 15% and 3%, respectively [2]. Therefore, early detection of pancreatic cancer without local invasion or distant metastasis is critical for improving the survival of patients with pancreatic cancer. However, early diagnosis of pancreatic cancer is difficult owing to nonspecific symptoms, with stage IA and IB cases accounting for only 1.8% and 2.3% of all cases, respectively [6]. Patients with pancreatic cancer who present with symptoms generally have advanced-stage disease, and 75% of patients with stage 0 and stage I pancreatic cancer are asymptomatic [7]. Therefore, it is important to detect pancreatic cancer in asymptomatic populations to improve the prognosis. However, to date, no blood biomarkers have been used to identify patients with pancreatic cancer at an early stage.

The US Preventive Services Task Force does not recommend pancreatic cancer screening in asymptomatic adults [8]. The low prevalence of pancreatic cancer makes the screening of asymptomatic adult populations unfeasible using existing diagnostic methods. Moreover, the existing diagnostic methods have unacceptably high rates of false positive findings [9]. However, high-risk populations with certain inherited genetic syndromes, a history of familial pancreatic cancer, or intraductal papillary neoplasms of the pancreas (IPMN) are recommended for pancreatic cancer screening [10, 11]. However, in the framework of an imaging-based pancreatic cancer surveillance research program in high-risk individuals, almost half of the individuals developed neoplastic lesions without prior signs on imaging, and by the time of detection or surgical treatment, most had already progressed beyond an early stage (T1 N0 M0) [12]. According to the Japanese nationwide calculation of cancer screening detection rates using abdominal ultrasound (AUS), only 152 pancreatic cancer cases were detected among 3,005,393 examinees (0.005%) [13]. Thus, more effective, and less invasive diagnostic tools for early-stage pancreatic cancer are urgently required.

MicroRNAs (miRNAs) are small non-coding RNA composed of 18–24 base pairs of single-chain molecules [14, 15]. MiRNAs modulate gene expression by decreasing target mRNA stability or repressing translational efficiency. They can stably exist in severe conditions, including in serum or pancreatic juice because some miRNAs are at least partly packaged into extracellular vesicles or included in an RNA-induced simple complex (RISC) with the Agonature2 protein to protect against the elimination of RNase [16–18]. Therefore, circulating miRNAs are potential novel targets for liquid biopsies. Some retrospective studies have shown that the expression of specific miRNAs in plasma or serum can distinguish patients with pancreatic cancer from healthy participants or patients with benign pancreatic diseases [19–30]. However, in most of these studies, the data were not validated using independent case-control cohorts. In addition, previous studies included only a small number of patients with early-stage (stage 0-I) pancreatic cancer. Furthermore, most of these studies analyzed only a few varieties of miRNAs, and not whole miRNA profiles. To the best of our knowledge, there have been few analyses of whole miRNA profiles for all samples in pancreatic cancer patients; however, these studies discriminated pancreatic cancer from other cancers but not from healthy controls [28], or the validation cohort included less than five samples from early-stage (stage 0-I) pancreatic cancer patients [31]. Importantly, its performance has not been validated in patients with asymptomatic early-stage (stage 0-I) pancreatic cancer.

In this study, to assess the ability of miRNA profiles and CA19-9 levels to distinguish subjects with pancreatic cancer at each stage from subjects without pancreatic cancer, we analyzed 425 serum samples, including 213 pancreatic cancer samples collected from 14 centers and 212 non-cancer healthy control samples collected from three centers. We comprehensively analyzed all miRNA profiles by next-generation sequencing (NGS) using an automated machine learning (AutoML) method to create discrimination models using highly expressed 100 miRNAs and CA19-9. The models were validated in an independent cohort study. Our data showed that the 100 highly expressed miRNAs and a combination of those with CA19-9 could be biomarkers for the specific and early detection of pancreatic cancer, and even for asymptomatic early-stage pancreatic cancer patients.

## Methods

### Patients and sample preparation

Serum samples were obtained from pancreatic cancer patients (*n* = 212) who were admitted or referred to Kyoto University Hospital (KUHP) (*n* = 56), Kindai University Hospital (KDUH) (*n* = 67), Kyoto Prefectural University of Medicine (KPUM) (*n* = 25), Hyogo Prefectural Amagasaki General Medical Center (AGMC) (*n* = 5), Japanese Red Cross Osaka Hospital (JRCOS) (*n* = 11), Japanese Red Cross Otsu Hospital (JRCOT) (*n* = 12), Japanese Red Cross Takatsuki Hospital (JRCT) (*n* = 3), Japanese Red Cross Wakayama Medical Center (JRCW) (*n* = 13), Kyoto Katsura Hospital (KKTR) (*n* = 4), Kobe City Nishi-Kobe Medical Center (KNMC) (*n* = 2), Kitano Hospital (KTNH) (*n* = 5), Shiga General Hospital (SGH) (*n* = 3), Shinko Hospital (SKHP) (*n* = 3), and Tenri Hospital (TNRH) (*n* = 11), all of which are secondary or tertiary care hospitals, between 2020 and 2023 and stored at −80 °C. The inclusion criteria were age over 20 years, histologically confirmed pancreatic cancer in a surgically resected specimen, or a computed tomography (CT) scan showing a solid mass in the pancreas or dilatation of pancreatic duct in patients not undergoing surgery with histology or cytology from the pancreas that confirmed the diagnosis of adenocarcinoma. The clinical stage of pancreatic cancer was determined using a CT scan according to the UICC 7th criteria. Patients with pancreatic cancer were randomly divided into a training cohort and a validation cohort under the following restrictions: 30 cases in stage 0-I, stage II, III, and IV were assigned to the validation cohort, and the remaining 92 pancreatic cancer cases were assigned to the training cohort.

A total of 213 serum samples from healthy individuals without cancer were collected from three independent cohorts. The three cohorts included volunteers aged over 40 years who were recruited from the OCROM clinic (OCROMC) (*n* = 71), Osaka Pharmacology Clinical Research Hospital (OPHACH) (*n* = 71), and ToCROM clinic (TOCROMC) (*n* = 71), all of which are primary care hospitals, in 2021 and stored at −80 °C. The inclusion criterion for healthy control participants was no history of malignant tumors according to self-reported medical history at the time of blood sampling and one year later. Healthy control participants were randomly divided into training and validation cohorts under the following restrictions: 120 cases were assigned to the validation cohort, and the remaining 93 healthy control participants were assigned to the training cohort.

### Blood sample collection and miRNA extraction from serum

Blood samples were collected into serum-separating tubes. After blood collection, the serum was separated by centrifugation and aliquoted into cryotubes. These sera were frozen at −80 °C until miRNA extraction. The time interval between blood sampling and serum freezing at −80 °C was observed strictly within the same day, and serum samples that showed hemolysis were excluded. RNA samples containing miRNA were extracted from the serum using the Maxwell® RSC miRNA Plasma and Serum kit (Promega, AS1680). QIAseq miRNA Library QC Spike-ins (Qiagen, 331535) was spiked into each serum sample to monitor RNA extraction. Concentrations of miRNAs were quantified using Qubit™ microRNA Assay Kits (Invitrogen, Q32881). These miRNA samples were stored at −80 °C until NGS library preparation.

### NGS library preparation and NGS measurement

miRNA libraries were prepared using the QIAseq miRNA Library Kit (Qiagen, 331509) and the QIAseq miRNA NGS 96 Index IL (96) (Qiagen, 331565) using Agilent Bravo NGS (Agilent Technologies, RRID:SCR_019473). The library size distribution was determined using a TapeStation HS D1000 system (Agilent Technologies, 5067-5584 and 5067-5585). Library samples were pooled and the NextSeq PhiX Control Kit (Illumina, FC-110-3002) was spiked into the sample mixture according to the manufacturer’s recommendations. The pooled samples were sequenced in four lanes of the NextSeq 500/550 High Output Kit v2.5 (75 cycles) (Illumina, 20024906) using the NextSeq 550 platform (Illumina, RRID:SCR_016381). The sequenced reads were annotated using the QIAseq miRNA primary analysis pipeline provided by the GeneGlobe Data Analysis Center (https://geneglobe.qiagen.com/jp/analyze/, RRID:SCR_021211). Sequencing outputs were mapped to miRBase v21 using the QIAseq miRNA Primary Analysis Pipeline.

### miRNA selection and normalization

We performed miRNA filtration using the read count data of 2588 miRNAs and 425 samples. Among the 2588 miRNAs, we excluded miRNAs if they did not meet the following criteria: the read count was one or more in all the samples. The remaining 230 miRNAs were normalized by ComBat (RRID:SCR_010974) in the R (version 4.2.3, RRID:SCR_001905) “sva” package and log2-transformed. These miRNAs were used for PCA and hierarchical unsupervised clustering analysis. The miRNAs are listed in Supplementary Table 1.

For the development of classification models, miRNAs were excluded if they did not meet the following criteria: a read count of 50 or more in all the samples. The ComBat-normalized and log2-transformed values of the remaining 100 miRNAs were used to construct discrimination models. These miRNAs are listed in Supplementary Table 2.

### Construction of pancreatic cancer discrimination models

To construct pancreatic cancer discrimination models, we utilized the AutoML platform DataRobot (version 8.0.12, Boston, Massachusetts) [32, 33]. DataRobot can automatically create more than 60 discrimination models and their combinations, called ensemble models. Using the expression data of 100 miRNAs, a 5-fold cross-validation (CV) AUC was calculated against 64 algorithms. This calculation was repeated 100 times and the division patterns of the training cohort were randomly changed for cross-validation. Eight algorithms with high average 5-CV AUC values were selected for the final miRNA model. For the miRNA model, the selected 8 algorithms were as follows: elastic net [34], light gradient boosting [35] (RRID:SCR_021697), nystroem kernel support vector machine [36], extremely randomized trees [37], Keras wide residual neural network [38], regularized logistic regression [39], stochastic gradient descent [40], and extreme gradient boosting [41] (RRID:SCR_021361). The average value of the eight prediction score outputs from each algorithm was used as the final miRNA index. For the miRNA+CA19-9 model, 5-CV AUC calculations and model selection were performed in the same way as described above, using 100 miRNA expression data points in combination with serum CA19-9 concentration data. For the miRNA+CA19-9 model, the selected 8 algorithms were as follows: RuleFit [42], gradient-boosted trees [43], gradient-boosted greedy trees [44], random forest [45], Keras wide residual neural network, elastic net, stochastic gradient descent, and extreme gradient boosting. The average value of the eight prediction score outputs from each algorithm was used as the final miRNA+CA19-9-index.

### Construction of pancreatic cancer discrimination models using data from Ion GeneStudio S5 system

For miRNA quantification using the Ion GeneStudio S5 Prime system (Thermo Fisher Scientific, RRID: SCR_017984), miRNA libraries were prepared using the QIAseq miRNA Library Kit and the QIAseq miRNA 48 Index TF (96) (Qiagen, 331585) using Agilent Bravo NGS. The pooled libraries were loaded into the Ion 540 Chip (Thermo Fisher Scientific, A27766) with the Ion Chef system (Thermo Fisher Scientific, 4484177) and sequenced with the Ion GeneStudio S5 Prime system. The sequenced reads were processed as for NextSeq 550. miRNA reads were normalized by reference-batch ComBat in the R (version 4.2.3) “sva” package. Pancreatic cancer discrimination models using data from the Ion GeneStudio S5 system were constructed using the same 100 miRNAs used in the models built on NextSeq 550 data. The same eight algorithms as the NextSeq 550-based models were used and the average of these prediction scores was used as the final index.

### Statistical analysis

PCA, t-SNE, UMAP, hierarchical unsupervised clustering analysis with heatmaps, box plots, scatter plots, confusion matrices, ROC curves, and AUC calculations were conducted using the statistical analysis software R (version 4.2.3). PCA, t-SNE, and UMAP analyses were conducted by prcomp (RRID:SCR_014676), Rtsne (perplexity = 30, as the default value, RRID:SCR_016342), and umap in the R “stats,” “Rtsne,” and “umap” packages, respectively. In each PCA, t-SNE, and UMAP mapping, 95% confidence ellipses were drawn by stat_ellipse in the R “ggplot2” package. Heatmap and dendrograms were drawn by Heatmap in the R “ComplexHeatmap” package. Student’s *t*-test for continuous variables and Pearson’s χ2 test for categorical variables were used to analyze patient characteristics of the training cohort and validation cohort. To evaluate model performance of sensitivity and specificity, 95% CIs were calculated using the bootstrap method by the R “pROC” package. To evaluate the influence of patient backgrounds of age, sex, drinking habits, history of smoking, blood collection centers, diabetes mellitus, and focal pancreatic parenchymal atrophy (FPPA) on constructed models, *p*-values of the Kolmogorov–Smirnov test were calculated by ks.test in the R “stats” package, and corrected by the Bonferroni method. To evaluate the correlation between miRNA index and CA19-9 level, and between miRNA index and tumor size, Spearman’s correlation coefficient was calculated by cor.test in the R “stats” packages. To evaluate AUC values of ROC curves with early-stage or asymptomatic pancreatic cancers of constructed models, *p*-values were calculated by roc.test in the R “pROC” package, and corrected by the Bonferroni method. To evaluate sensitivities of early-stage or asymptomatic pancreatic cancers of constructed models, *p*-values of the McNemar test were calculated by mcnemar.test in the R “stats” package, and corrected by the Bonferroni method.

## Results

### Study design

**Fig. 1:**
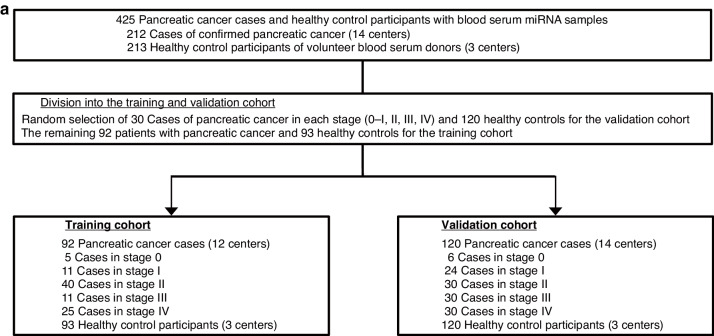
The workflow of pancreatic cancer patients and healthy control participants for developing and evaluating prediction models. **a** All the 425 serum samples, including 213 pancreatic cancer patients from 14 hospitals and 212 healthy controls without cancer from three clinics, were randomly divided into the training and validation cohorts.

A total of 425 serum samples, including those of 213 pancreatic cancer patients from 14 hospitals and of 212 healthy controls without cancer from three clinics, were analyzed by miRNA sequencing to generate global miRNA expression profiles. According to the UICC 7th edition criteria, we subdivided pancreatic cancers based on the clinical stage: stage 0, stage I, stage II, stage III, and stage IV. Serum samples were collected prior to treatment. To validate the diagnostic performance in patients with pancreatic cancer in each stage, we randomly selected 30 pancreatic cancer cases in stage 0-I, stage II, III, and IV, and 120 healthy control cases as a validation cohort. The remaining 92 patients with pancreatic cancer and 93 non-cancer healthy controls were assigned to the training cohort (Fig. 1).

Participants’ characteristics are presented in Table 1. Owing to restrictions in the sampling of healthy participants, there was a significant difference in age, drinking habits, and diabetes mellitus status in the training cohort and in sex, age, history of smoking, and diabetes mellitus in the validation cohort (*P* < 0.05) between patients with pancreatic cancer and healthy participants. Owing to the random selection of 30 pancreatic cancer cases at each stage, there was a significant difference in stage (*P* < 0.05) (Supplementary Table 3) between the validation and training cohorts in pancreatic cancer patients.

**Table 1:** The characteristics of pancreatic cancer patients and healthy controls in the training and validation cohorts and the *p*-value of pancreatic cancers vs. healthy controls in each cohort.

	Training		Validation	
	PC	HC	PC	HC
Total [no.]	92	93	120	120
Sex				
Male [no.]	57	51	73	55
Female [no.]	35	42	47	65
*P*-value (PC vs HC)	0.405^b^		0.028^b^	
Age				
Median (range) [year]	74 (31–92)	62 (40–88)	72.5 (38–86)	61.5 (40–86)
*P*-value (PC vs HC)	<0.001^a^		<0.001^a^	
History of smoking				
Current [no.]	18	12	17	10
Former [no.]	36	26	53	36
Never [no.]	38	55	50	74
*P*-value (PC vs HC)	0.052^b^		0.008^b^	
Drinking habits				
Everyday [no.]	16	13	33	26
Sometimes [no.]	16	36	26	37
Non [no.]	60	44	60	57
NA [no.]	0	0	1	0
*P*-value (PC vs HC)	0.005^b^		0.244^b^	
Diabetes mellitus				
No [no.]	60	78	73	102
Yes [no.]	32	15	47	18
*P*-value (PC vs HC)	0.006^b^		<0.001^b^	
CA19-9				
Median (range) [U/mL]	156.8 (0.4–6.8 × 10^4^)	4.9 (2.0–77.1)	83.3 (0.6–1.2 × 10^5^)	4.7 (2.0–38.1)
NA [no.]	0	0	0	4
*P*-value (PC vs HC)	0.019^a^		0.013^a^	
CEA				
Median (range) [ng/mL]	3.5 (0.7–3.1 × 10^2^)	1.7 (0.6–9.1)	3.9 (0.6–1.7 × 10^3^)	1.7 (0.6–47)
NA [no.]	0	0	0	4
*P*-value (PC vs HC)	0.024^a^		0.087^a^	
DUPAN-2				
Median (range) [U/mL]	115 (25–1.4 × 10^5^)	25 (25–92)	152.2 (25–3.1 × 10^7^)	25 (25–98)
NA [no.]	14	0	20	4
*P*-value (PC vs HC)	0.046^a^		0.317^a^	
Stage (asymptomatic, symptomatic, NA) [no.]				
0	5 (4, 1, 0)	-	6 (5, 1, 0)	-
I	11 (5, 3, 3)	-	24 (16, 5, 3)	-
II	40 (3, 8, 29)	-	30 (1, 4, 25)	-
III	11 (2, 0, 9)	-	30 (2, 3, 25)	-
IV	25 (2, 5, 18)	-	30 (2, 6, 22)	-

*PC* pancreatic cancer, *HC* healthy control, *NA* not available.

^a^Student’s *t*-test.

^b^Pearson’s chi-squared test.

### The comprehensive analysis of the miRNA expression profiles identified differences in patients with pancreatic cancer and healthy participants

**Fig. 2:**
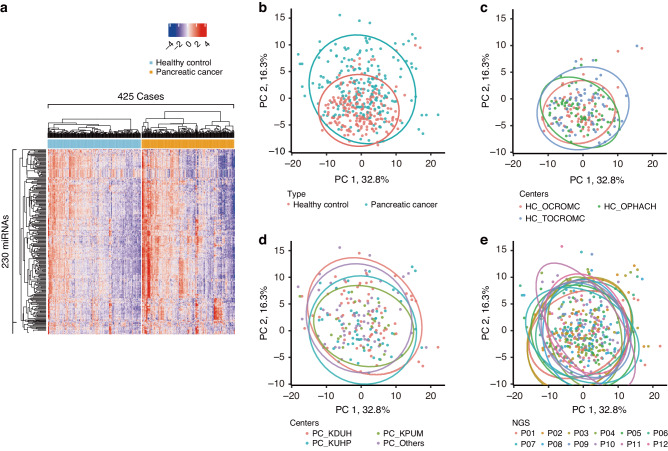
The serum miRNA expression profiles in healthy controls differed from those in pancreatic cancer patients. **a** Heatmap representing hierarchical unsupervised clustering analysis of the miRNA sequencing of healthy controls (blue) and pancreatic cancer patients (orange). **b** Principal component analysis (PCA) of the miRNA sequencing of healthy controls (blue) and pancreatic cancer patients (orange). **c**–**e** The PCA grouped by hospitals, clinics, and the NGS measurements. HC healthy control, PC pancreatic cancer.

We examined the expression levels of 2588 miRNAs comprehensively for miRNA sequencing with NextSeq 550 (Illumina). First, we focused on the 230 miRNAs commonly detected in all 425 samples. For these miRNAs, we performed hierarchical unsupervised clustering analysis with a heatmap (Fig. 2a) and principal component analysis (PCA) mapping (Fig. 2b) to visualize the expression patterns of miRNAs in all samples in the training and validation cohorts. Another clustering result, without the separation of healthy controls and patients with pancreatic cancer, also showed that each sample type formed clusters (Supplementary Fig. 1A). These classification analyses indicated that the miRNA expression profiles in non-cancer controls were relatively distinct from those in patients with pancreatic cancer. Furthermore, the PCA revealed no significant differences in patients with pancreatic cancer among hospitals, in healthy controls among clinics, and in all the samples among the NGS measurements (Fig. 2c–e). Similar analyses were conducted using the t-distributed stochastic neighbor embedding (t-SNE), and the uniform manifold approximation and projection (UMAP) methods. These results also supported differences in miRNA expression profiles between healthy controls and patients with pancreatic cancer, but no significant differences among the blood collection centers and the NGS measurements (Supplementary Fig. 2A–H).

### We created the best discrimination models using AutoML in the training cohort and validated the performance in the independent validation cohort

**Fig. 3:**
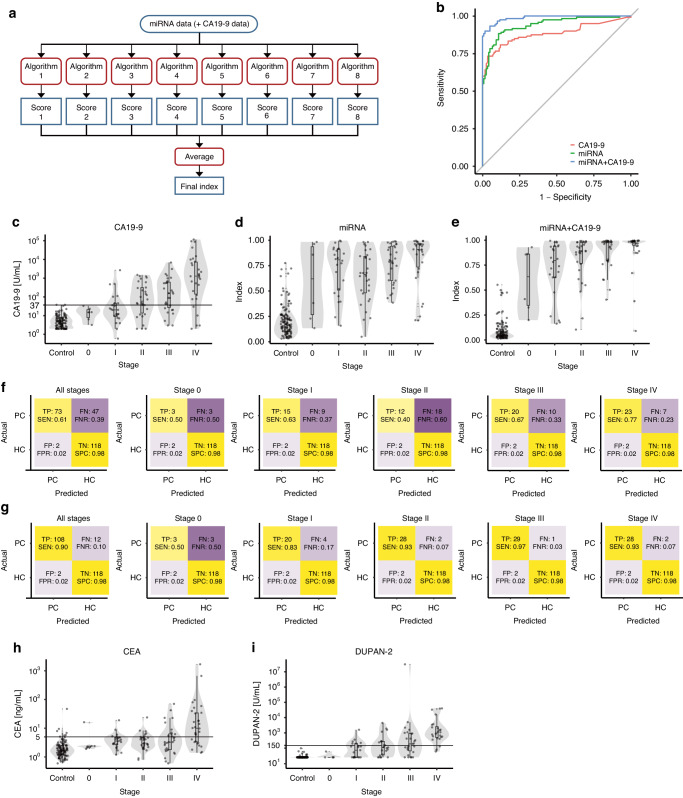
Creation of the best discrimination models using AutoML in the training cohort and validation in the independent validation cohort. **a** Schematic procedure of the index calculation in miRNA model and miRNA+CA19-9 model. **b** ROC curve for the performance of serum CA19-9 alone (red), miRNA model (green), and miRNA+CA19-9 model (blue) in the validation cohort. **c**–**e** Box plots of CA19-9, indices of miRNA model, and miRNA+CA19-9 model of healthy participants and pancreatic cancer patients in each stage (0, I, II, III, and IV) in the validation cohort. **f**, **g** Confusion matrices of miRNA model and miRNA+CA19-9 model at 98% specificity in each stage of the validation cohort. TP True Positive, TN True Negative, FP False Positive, FN False Negative, SEN Sensitivity, SPC Specificity, FPR False Positive Rate, FNR False Negative Rate. The color gradient indicated the rate of each metric. **h**, **i** Box plots of CEA and DUPAN-2 of healthy participants and pancreatic cancer patients in each stage (0, I, II, III, and IV) in the validation cohort.

Next, using the training cohort, diagnostic models were constructed to discriminate patients with pancreatic cancer from healthy controls. To this end, we focused on the highly and robustly expressed miRNAs. One hundred highly expressed miRNAs were used to create a diagnostic model in the training cohort using the AutoML platform DataRobot. We used eight algorithms to calculate the prediction scores and took the average of the prediction scores produced by the eight algorithms to create the best discrimination model (Fig. 3a), designated as the miRNA model, which exhibited the best discrimination performance in the training cohort. We also created a diagnostic model using the 100 highly and robustly expressed miRNAs in combination with serum CA19-9 in the same way as creating the miRNA model, designated as the miRNA+CA19-9 model, which exhibited the best discrimination performance in the training cohort.

Next, we evaluated the diagnostic performance of serum CA19-9, the miRNA model, and the miRNA+CA19-9 model in an independent validation cohort. Receiver operating characteristic (ROC) curves for serum CA19-9, the miRNA model, and the miRNA+CA19-9 model in the validation cohort revealed that both the miRNA model and the miRNA+CA19-9 model showed better discrimination performance than conventional serum CA19-9 in the validation cohort (Fig. 3b). The miRNA and miRNA+CA19-9 models exhibited high indices for patients with pancreatic cancer, even in the early stages. Although serum CA19-9 levels gradually increased as the patient’s disease stages progressed, positive CA19-9 levels in the validation cohort were observed only in 29% of patients in stage I, while none of the patients in stage 0 had a positive level (Fig. 3c and Table 2). In contrast, the miRNA and miRNA+CA19-9 models successfully discriminated healthy controls from patients with pancreatic cancer in stages II-IV as well as in stage 0-I (Fig. 3d–g and Table 2).

**Table 2:** The performance of serum CA19-9, miRNA model, and miRNA+CA19-9 model to discriminate pancreatic cancer patients from healthy participants.

	CA19-9	miRNA model				miRNA+CA19-9 model			
AUC	0.88	0.94				0.99			
95% CI	0.84–0.93	0.91–0.97				0.98–1.00			
Specificity	0.98^a^	0.85	0.90	0.95	0.98^b^	0.85	0.90	0.95	0.98^b^
95% CI	0.96–1.00	-	-	-	-	-	-	-	-
All stages sensitivity	0.60	0.91	0.84	0.78	0.61	0.98	0.97	0.93	0.90
95% CI	0.51–0.68	0.82–0.96	0.75–0.93	0.59–0.88	0.50–0.81	0.94–1.00	0.90–0.99	0.86–0.98	0.83–0.95
Each stage sensitivity									
Stage 0	0.00	0.67	0.50	0.50	0.50	1.00	1.00	0.83	0.50
95% CI	0.00–0.00	0.33–1.00	0.17–1.00	0.17–0.83	0.17–0.83	1.00–1.00	0.50–1.00	0.17–1.00	0.17–1.00
Stage I	0.29	0.92	0.83	0.75	0.63	0.96	0.92	0.83	0.83
95% CI	0.13–0.50	0.75–1.00	0.67–1.00	0.54–0.92	0.42–0.83	0.88–1.00	0.71–1.00	0.67–1.00	0.63–0.96
Stage II	0.63	0.87	0.80	0.73	0.40	0.97	0.97	0.93	0.93
95% CI	0.47–0.80	0.70–0.97	0.63–0.97	0.37–0.90	0.23–0.77	0.90–1.00	0.87–1.00	0.83–1.00	0.80–1.00
Stage III	0.70	1.00	0.97	0.87	0.67	1.00	1.00	1.00	0.97
95% CI	0.53–0.87	0.97–1.00	0.80–1.00	0.60–1.00	0.47–0.93	1.00–1.00	1.00–1.00	0.90–1.00	0.87–1.00
Stage IV	0.83	0.90	0.83	0.83	0.77	0.97	0.97	0.97	0.93
95% CI	0.70–0.97	0.73–1.00	0.70–0.97	0.67–0.97	0.60–0.93	0.90–1.00	0.90–1.00	0.87–1.00	0.83–1.00

^a^ Threshold = 37 U/mL.

^b^The same specificity as that of CA19-9.

In the independent validation cohort, the AUC was 0.88 (95% confidence interval (CI), 0.84–0.93) for serum CA19-9, 0.94 (95% CI, 0.91–0.97) for the miRNA model, and 0.99 (95% CI, 0.98–1.00) for the miRNA+CA19-9 model when patients with pancreatic cancer were tested against healthy participants (Table 2).

We also evaluated the diagnostic performance of serum CEA and DUPAN-2, other pancreatic cancer markers. Serum CEA and DUPAN-2 levels, similar to CA19-9, gradually increased as the patients’ disease status progressed however, only 20% of patients in stages 0 and I were positive for each tumor marker in the validation cohort (Fig. 3h, i). We investigated the influence of patient background, including age, sex, drinking habits, smoking history, blood collection center, diabetes mellitus status, FPPA detection, tumor size, and CA19-9 level. No association was found between discrimination scores and age, sex, drinking habits, history of smoking, blood collection center, or diabetes mellitus status, FPPA detection, tumor size, or CA19-9 level. (Supplementary Figs. 3A–J and 4A–G).

To validate the reproducibility of diagnostic performance between miRNA measurement platforms, we measured miRNA profiles with Ion GeneStudio S5 and predicted pancreatic cancer or healthy control in the validation cohort on models constructed by miRNA profiles from NextSeq 550. Validation results using data from the Ion GeneStudio S5 system were comparable to those of NextSeq 550, confirming the robustness of the models (Supplementary Fig. 6, Supplementary Table 6).

### The miRNA+CA19-9 model showed the highest performance in discriminating patients with pancreatic cancer in stage 0-I or stage 0-II from healthy controls

**Fig. 4:**
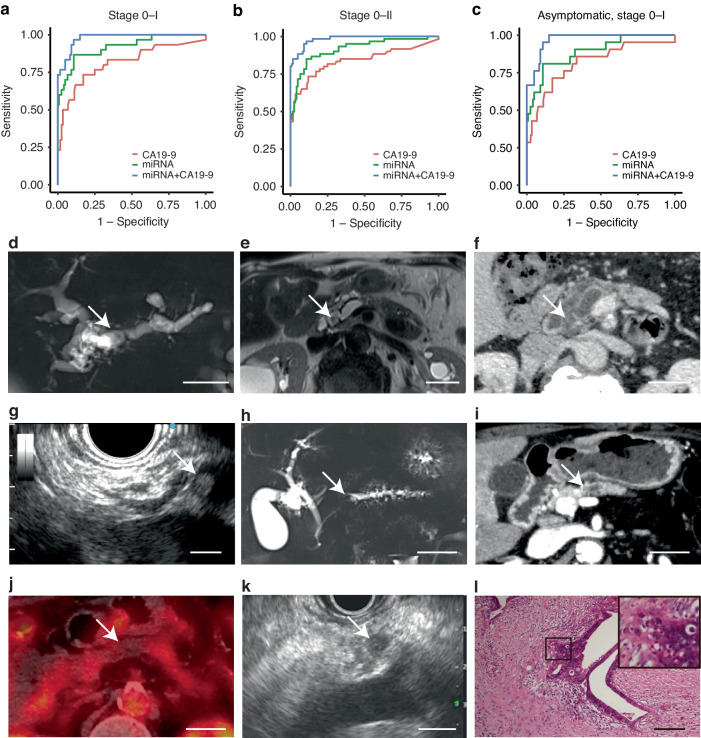
The 100 highly expressed miRNAs and CA19-9 in blood serum successfully discriminated healthy controls from patients with pancreatic cancer, not only in the advanced stage but also in the early stage (stage 0-I) with high sensitivity and specificity. **a**, **b** ROC curves for the performance of serum CA19-9 alone (red), miRNA model (green), and miRNA+CA19-9 model (blue) in discriminating patients with pancreatic cancer in stage 0-I (**a**) and stage 0-II (**b**) from healthy controls. **c** ROC curves for the performance of serum CA19-9 alone (red), miRNA model (green), and miRNA+CA19-9 model (blue) in discriminating asymptomatic patients with pancreatic cancer in stage 0-I in the validation cohort. **d**–**l** Asymptomatic patients with pancreatic cancer in stage I: case 1 (**d**–**g**) and case 2 (**h**–**l**). **d** MRI revealed main pancreatic duct dilatation (arrow). Scale bar, 3.0 cm (**e**) MRI revealed a low-signal area in the main pancreatic duct on T2-weighted images (arrow). Scale bar, 3.0 cm (**f**) Contrast-enhanced CT revealed a slightly enhanced tumor in the main pancreatic duct (arrow). Scale bar, 3.0 cm (**g**) EUS revealed a mass, 5 mm in diameter, in the main pancreatic duct (arrow). Scale bar, 0.50 cm (**h**) MRI revealed main pancreatic duct dilatation (arrow). Scale bar, 3.0 cm (**i**) Contrast-enhanced CT revealed main pancreatic dilatation (arrow). A tumor could not be detected by CT. Scale bar, 3.0 cm (**j**) A tumor could not be detected in PET-CT (arrow). Scale bar, 3.0 cm (**k**) A tumor, 6 mm in diameter, was detected by EUS (arrow). Scale bar, 1.0 cm (**l**) Hematoxylin, and eosin staining of the surgical specimen revealed pancreatic cancer in stage IA. The inset shows invasive pancreatic cancer. Scale bar, 200 µm

We also evaluated the performance of each prediction model in discriminating patients with pancreatic cancer in stage 0-I or stage 0-II from healthy controls in the validation cohort. Among the three models, the miRNA+CA19-9 model showed the highest AUC in the three models. The diagnostic performance of the miRNA model was higher than that of CA19-9 in patients with stage 0-I (Fig. 4a) and stage 0-II (Fig. 4b) pancreatic cancers compared to healthy participants. The AUC was 0.81 (95% CI, 0.71–0.92) and 0.84 (95% CI, 0.76–0.91) for serum CA19-9, 0.92 (95% CI, 0.86–0.98) and 0.92 (95% CI, 0.87–0.96) for the miRNA model, and 0.98 (95% CI, 0.96–1.00) and 0.98 (95% CI, 0.97–1.00) for the miRNA+CA19-9 model when patients with pancreatic cancer in stage 0-I or stage 0-II were tested against healthy participants, respectively (Supplementary Table 4).

The AUC of the miRNA+CA19-9 model was significantly higher than that of CA19-9 in patients with pancreatic cancer in stage 0-I (Fig. 4a) and stage 0-II (Fig. 4b) in the validation cohort (*P* < 0.05). The sensitivities of the miRNA and miRNA+CA19-9 models at 98% specificity, the same as that of CA19-9, were significantly higher than that of CA19-9 in patients with pancreatic cancer in stage 0-I tested against healthy participants in the validation cohort (*P* < 0.05).

These results indicated that the 100 highly expressed miRNAs and CA19-9 in blood serum successfully discriminated healthy controls from patients with pancreatic cancer, not only in the advanced stage but also in the early stage (stage 0-I) with high sensitivity and specificity.

To further determine whether these discrimination models are useful for asymptomatic patients with pancreatic cancer in the early stage (stage 0-I), we investigated the performance of each model in 21 cases of asymptomatic patients with pancreatic cancer in stage 0-I. The ROC curves for serum CA19-9, the miRNA model, and the miRNA+CA19-9 model using the validation cohort revealed that both the miRNA and miRNA+CA19-9 models showed better discrimination performance than conventional serum CA19-9 in asymptomatic patients with pancreatic cancer in stage 0-I (Fig. 4c). The AUC was 0.81 (95% CI, 0.70–0.93) for serum CA19-9, 0.89 (95% CI, 0.81–0.97) for the miRNA model, and 0.97 (95% CI, 0.95–.00) for the miRNA+CA19-9 model when asymptomatic patients with pancreatic cancer of stage 0-I were tested against healthy participants in the validation cohort. The AUC and sensitivity of the miRNA+CA19-9 model at 98% specificity were significantly higher than those of CA19-9 (*P* < 0.05) (Supplementary Table 5).

These results indicate that the 100 highly expressed miRNAs and their combination with CA19-9 could be used as biomarkers for screening asymptomatic patients with pancreatic cancer in stage 0-I.

Finally, we present representative cases in which the miRNA and miRNA+CA19-9 models could discriminate asymptomatic patients with early-stage pancreatic cancer from healthy controls.

Case 1: An asymptomatic male in his 70 s visited Kyoto University Hospital for a routine check-up for dilatation of the main pancreatic duct and a pancreatic cyst. Magnetic resonance imaging (MRI) revealed worsening of the dilatation of the main pancreatic duct, cyst enlargement, and a low-signal area in the main pancreatic duct on T2-weighted images (Fig. 4d, e). The patient underwent contrast-enhanced CT, contrast-enhanced endoscopic ultrasound (EUS), serum tumor marker assessments, and serum miRNA sequencing. A tumor, 5 mm in diameter, in the main pancreatic duct was detected on contrast-enhanced CT and EUS (Fig. 4f, g). CA19-9 of 12.1, CEA of 2.3, the miRNA index of 0.92, and the miRNA+CA19-9 index of 0.66 indicated negative, negative, positive, and positive, respectively. Pancreatic juice cytology revealed adenocarcinoma, and the patient was diagnosed with pancreatic cancer at clinical stage I. The patient underwent surgery and was pathologically diagnosed with stage IB pancreatic cancer.

Case 2: An asymptomatic male in his 70 s visited Kyoto University Hospital because of main pancreatic duct dilatation detected on MRI screening (Fig. 4h). The patient underwent AUS, contrast-enhanced CT, positron emission tomography-CT (PET-CT), EUS (Fig. 4i–k), serum tumor marker measurements, and serum miRNA sequencing. Dilatation of the main pancreatic duct was detected in CT, MRI, and EUS. A tumor could not be detected by AUS, CT, MRI, or PET-CT but could only be detected by EUS. CA19-9 of 20.5, CEA of 4.2, the miRNA index of 0.45, and the miRNA+CA19-9 index of 0.68 indicated negative, negative, negative, and positive, respectively. Histological examination of EUS-FNA samples from the tumor revealed adenocarcinoma, and the patient was diagnosed with clinical stage I pancreatic cancer. The patient underwent surgery and was pathologically diagnosed with stage IA pancreatic cancer (Fig. 4l).

## Discussion

Early diagnosis of pancreatic cancer is difficult because of nonspecific symptoms, with stage I cases accounting for only 4.1% [7] of all pancreatic cancer cases. Sensitive and specific biomarkers for identifying patients with early-stage pancreatic cancer are urgently required. In this study, we comprehensively analyzed serum miRNA profiles using NGS of all serum samples from patients with pancreatic cancer and healthy controls in a large sample set (*N* = 425) collected from 17 centers, including 46 patients with pancreatic cancer in stage 0-I. The discrimination models generated using the training cohort were validated using an independent validation cohort (*N* = 240). High diagnostic accuracy (AUC:0.98) was validated even in an independent early-stage (stage 0-I) pancreatic cancer cohort (*N* = 30), which included 21 patients with asymptomatic pancreatic cancer. Using an ensemble model that combines machine learning algorithms, our model has good discriminative ability and high robustness.

Liu et al. [27] and Nakamura et al. [30] recently reported that a combination of miRNAs in plasma with serum CA19-9 can discriminate patients with pancreatic cancer from healthy controls with high sensitivity and specificity (AUC of 0.98 and 0.99, respectively). However, these prediction models have not been validated using an independent validation cohort [27, 30]. In this study, we validated our pancreatic cancer prediction models, the miRNA model and the miRNA+CA19-9 model, with an independent validation cohort to differentiate patients with pancreatic cancer from healthy controls.

One of the greatest strengths of this study was its ability to discriminate patients with pancreatic cancer in the early stage (stage 0-I) from healthy controls with high sensitivity and specificity. By comprehensively analyzing all the miRNA profiles and using AutoML, our miRNA+CA19-9 model showed the highest performance in terms of AUC for discriminating patients with pancreatic cancer from healthy controls compared with all previously reported miRNA studies in which the performance was validated in independent cohorts containing over 10 patients with stage 0-I pancreatic cancer [30]. Furthermore, the high performance of our discrimination models was validated in 21 asymptomatic patients with pancreatic cancer in stage 0-I. These results suggest that the 100 highly expressed miRNAs and their combination with CA19-9 could be biomarkers for detecting asymptomatic patients with pancreatic cancer in stage 0-I.

Most of the 100 miRNAs used in our discrimination models were dismissed in previous studies. This is possibly because previous studies focused only on miRNAs that were differentially expressed between patients with pancreatic cancer and controls, and constructed discrimination models using them. We extracted highly and robustly expressed miRNAs and used them for model construction, regardless of differences in expression levels.

Another strength of this study is that we used large-scale miRNA sequencing data from 425 serum samples using an ensemble model that combined machine learning algorithms to identify the best combination of miRNAs for pancreatic cancer screening. Compared to conventional cancer discriminations with miRNA, which sets a threshold on the expression levels of a small number of miRNAs added together [29], combining a large number of miRNAs through machine learning improves the discrimination ability of pancreatic cancer. A possible disadvantage of machine learning is that the time cost of creating models is high; however, AutoML can build models faster.

This study had a few limitations. First, the outcome was confounded by age because healthy volunteer blood donors were younger than the patients with pancreatic cancer. However, separate analyses of cases and controls revealed no correlation between age, drinking habits, smoking history, miRNA index, and the miRNA+CA19-9 index. These findings suggest that the models may be practical, independent of age, drinking habits, and smoking history, even under more realistic clinical conditions. Second, we only assessed patients with pancreatic cancer and healthy participants retrospectively. This limitation raises the question of whether these discrimination models are useful for other populations, especially high-risk populations with certain inherited genetic syndromes, a history of familial pancreatic cancer, or IPMN, which are precancerous lesions of pancreatic cancer. Therefore, future prospective screening studies involving high-risk populations and asymptomatic adults are required to successfully translate these findings to clinical settings. Third, it is unknown whether this miRNA model is sensitive to patients with other cancers, such as biliary cancer and colon cancer. In the future, it would be ideal to create diagnostic miRNA models of other cancers and to discriminate cancer patients from healthy controls using diagnostic discrimination models after comprehensive miRNA sequencing. Fourth, it is unclear whether the serum 100 miRNAs are produced from pancreatic cancer cells and how the 100 miRNAs function in pancreatic cancer. A previous report showed that miR-155, miR-181a, miR-181b, miR-21, miR-221, and miR-222 were upregulated in pancreatic cancer tumor, as confirmed by microarray and qPCR [46]. We examined if the reported miRNAs upregulated in the tumor were also upregulated in the serum. Some miRNAs (hsa-miR-181b-5p, hsa-miR-21-5p, and hsa-miR-222-3p) in the serum were slightly upregulated, while others were not in patients with pancreatic cancer compared with healthy controls. One possible reason is that the serum miRNA did not necessarily originate from the tumor tissue (Supplementary Fig. 5A–F). Fifth, the miRNA profile is affected by quantification methods such as library preparation protocol and measurement platform [47–49]. We investigated whether the high discrimination performance can be delivered using miRNA profiles measured by different platforms. Notably, our data suggested that the miRNA and miRNA+CA19-9 models constructed in this study robustly discriminated even when miRNA profiles were measured on Ion GeneStudio S5, a platform other than the NextSeq 550 used to create the models (Supplementary Fig. 6, Supplementary Table 6). There are three possible reasons for the robustness. First, both NextSeq 550 and Ion GeneStudio S5 accurately measured miRNA. Second, library preparation was highly automated. Third, we selected high-performance models from numerous blueprints of models and ensembled them with autoML. Furthermore, to validate the generality of the machine learning diagnostic model of pancreatic cancer with comprehensive miRNA expression profiles, we also created a model using miRNA profiles measured with the Ion GeneStudio S5 system. We found the model has a high performance comparable to that of the Illumina sequencing platform (Supplementary Fig. 7, Supplementary Table 7).

In conclusion, by comprehensively analyzing all miRNA profiles and using AutoML, the 100 highly expressed miRNAs and a combination of those with CA19-9 could discriminate patients with pancreatic cancer, even in the early stages (stage 0-I), from healthy controls. High diagnostic accuracy (AUC:0.98) was validated in an independent early-stage (stage 0-I) pancreatic cancer cohort, which included 21 asymptomatic patients. Our data demonstrate that the 100 highly expressed miRNAs and their combination with CA19-9 could be used as biomarkers for the specific and early detection of pancreatic cancer. We plan to prospectively study the utility of these discriminatory models for high-risk populations of pancreatic cancer.

## Supplementary information

supplemental Table 1

supplemental Table 2

supplemental Table 3

supplemental Table 4

supplemental Table 5

supplemental Table 6

supplemental Table 7

supplemental Figures

## Data Availability

The main data supporting the results of this study are available in this paper and in the Supplementary Information. The raw and analyzed datasets generated during the study are available for research purposes from the corresponding authors upon reasonable request. They also contain personal and patient data and are available for research purposes pending the completion of adequate paperwork, ensuring personal data protection, and ethical approval.

## References

[1] Rahib L, Smith BD, Aizenberg R, Rosenzweig AB, Fleshman JM, Matrisian LM. Projecting cancer incidence and deaths to 2030: the unexpected burden of thyroid, liver, and pancreas cancers in the United States. Cancer Res. 2014;74:2913–21. 24840647 10.1158/0008-5472.CAN-14-0155

[2] Siegel RL, Miller KD, Wagle NS, Jemal A. Cancer statistics, 2023. CA Cancer J Clin. 2023;73:17–48. 36633525 10.3322/caac.21763

[3] Hidalgo M. Pancreatic cancer. N Engl J Med. 2010;362:1605–17. 20427809 10.1056/NEJMra0901557

[4] Park W, Chawla A, O’Reilly EM. Pancreatic cancer: a review. JAMA. 2021;326:851–62. 34547082 10.1001/jama.2021.13027 PMC9363152

[5] Egawa S, Toma H, Ohigashi H, Okusaka T, Nakao A, Hatori T et al. Japan Pancreatic Cancer Registry; 30th Year Anniversary Japan Pancreas Society. 2012 www.pancreasjournal.com. 10.1097/MPA.0b013e318258055c 22750974

[6] Blackford AL, Canto MI, Klein AP, Hruban RH, Goggins M. Recent trends in the incidence and survival of stage 1A pancreatic cancer: a surveillance, epidemiology, and end results analysis. J Natl Cancer Inst. 2020;112:1162–9. 31958122 10.1093/jnci/djaa004 PMC7669234

[7] Kanno A, Masamune A, Hanada K, Maguchi H, Shimizu Y, Ueki T, et al. Multicenter study of early pancreatic cancer in Japan. Pancreatology. 2018;18:61–67. 29170051 10.1016/j.pan.2017.11.007

[8] Owens DK, Davidson KW, Krist AH, Barry MJ, Cabana M, Caughey AB, et al. Screening for pancreatic cancer: US preventive services task force reaffirmation recommendation statement. JAMA. 2019;322:438–44. 31386141 10.1001/jama.2019.10232

[9] Hart PA, Chari ST. Is screening for pancreatic cancer in high-risk individuals one step closer or a fool’s errand? Clin Gastroenterol Hepatol. 2019;17:36–38. 30268560 10.1016/j.cgh.2018.09.024 PMC6557281

[10] Goggins M, Overbeek KA, Brand R, Syngal S, Del Chiaro M, Bartsch DK, et al. Management of patients with increased risk for familial pancreatic cancer: updated recommendations from the International Cancer of the Pancreas Screening (CAPS) Consortium. Gut. 2020;69:7–17. 31672839 10.1136/gutjnl-2019-319352 PMC7295005

[11] Del Chiaro M, Besselink MG, Scholten L, Bruno MJ, Cahen DL, Gress TM, et al. European evidence-based guidelines on pancreatic cystic neoplasms. Gut. 2018;67:789–804. 29574408 10.1136/gutjnl-2018-316027 PMC5890653

[12] Overbeek KA, Goggins MG, Dbouk M, Levink IJM, Koopmann BDM, Chuidian M, et al. Timeline of development of pancreatic cancer and implications for successful early detection in high-risk individuals. Gastroenterology. 2022;162:772–785.e4. 34678218 10.1053/j.gastro.2021.10.014

[13] Ashida R, Tanaka S, Yamanaka H, Okagaki S, Nakao K, Fukuda J et al. The role of transabdominal ultrasound in the diagnosis of early stage pancreatic cancer: review and single-center experience. Diagnostics. 2019;9. 10.3390/diagnostics9010002. 10.3390/diagnostics9010002 PMC6468797 30587766

[14] Bartel DP. Review MicroRNAs: genomics, biogenesis, mechanism, and function. Cell 2004;116:281–97. 10.1016/s0092-8674(04)00045-5 14744438

[15] Ruan K, Fang X, Ouyang G. MicroRNAs: novel regulators in the hallmarks of human cancer. Cancer Lett. 2009;285:116–26. 19464788 10.1016/j.canlet.2009.04.031

[16] Kosaka N, Iguchi H, Ochiya T. Circulating microRNA in body fluid: a new potential biomarker for cancer diagnosis and prognosis. Cancer Sci. 2010;101:2087–92. 20624164 10.1111/j.1349-7006.2010.01650.x PMC11159200

[17] Kosaka N, Yoshioka Y, Fujita Y, Ochiya T. Versatile roles of extracellular vesicles in cancer. J Clin Investig. 2016;126:1163–72. 26974161 10.1172/JCI81130 PMC4811151

[18] Mlcochova H, Hezova R, Stanik M, Slaby O. Urine microRNAs as potential noninvasive biomarkers in urologic cancers. Urol Oncol. 2014;32:41.e1–41.e9. 24035473 10.1016/j.urolonc.2013.04.011

[19] Wang C, Wang J, Cui W, Liu Y, Zhou H, Wang Y, et al. Serum exosomal mirna-1226 as potential biomarker of pancreatic ductal adenocarcinoma. Onco Targets Ther. 2021;14:1441–51. 33664577 10.2147/OTT.S296816 PMC7924134

[20] Kim MW, Koh H, Kim JY, Lee S, Lee H, Kim Y, et al. Tumor-specific miRNA signatures in combination with ca19−9 for liquid biopsy-based detection of PDAC. Int J Mol Sci. 2021. 10.3390/ijms222413621. 10.3390/ijms222413621 PMC8703833 34948417

[21] Guo S, Qin H, Liu K, Wang H, Bai S, Liu S et al. Blood small extracellular vesicles derived miRNAs to differentiate pancreatic ductal adenocarcinoma from chronic pancreatitis. Clin Transl Med. 2021;11. 10.1002/ctm2.520. 10.1002/ctm2.520 PMC8431442 34586739

[22] Lai X, Wang M, McElyea SD, Sherman S, House M, Korc M. A microRNA signature in circulating exosomes is superior to exosomal glypican-1 levels for diagnosing pancreatic cancer. Cancer Lett. 2017;393:86–93. 28232049 10.1016/j.canlet.2017.02.019 PMC5386003

[23] Wu L, Zhou WB, Zhou J, Wei Y, Wang HM, Liu X, De. et al. Circulating exosomal microRNAs as novel potential detection biomarkers in pancreatic cancer. Oncol Lett. 2020;20:1432–40. 32724386 10.3892/ol.2020.11691 PMC7377032

[24] Goto T, Fujiya M, Konishi H, Sasajima J, Fujibayashi S, Hayashi A, et al. An elevated expression of serum exosomal microRNA-191, − 21, −451a of pancreatic neoplasm is considered to be efficient diagnostic marker. BMC Cancer. 2018;18. 10.1186/s12885-018-4006-5. 10.1186/s12885-018-4006-5 PMC5793347 29385987

[25] Khan IA, Rashid S, Singh N, Rashid S, Singh V, Gunjan D, et al. Panel of serum miRNAs as potential non-invasive biomarkers for pancreatic ductal adenocarcinoma. Sci Rep. 2021;11. 10.1038/s41598-021-82266-5. 10.1038/s41598-021-82266-5 PMC7854650 33531550

[26] Cote GA, Gore AJ, McElyea SD, Heathers LE, Xu H, Sherman S, et al. A pilot study to develop a diagnostic test for pancreatic ductal adenocarcinoma based on differential expression of select miRNA in plasma and bile. Am J Gastroenterol. 2014;109:1942–52. 25350767 10.1038/ajg.2014.331 PMC4261139

[27] Liu J, Gao J, Du Y, Li Z, Ren Y, Gu J, et al. Combination of plasma microRNAs with serum CA19-9 for early detection of pancreatic cancer. Int J Cancer. 2012;131:683–91. 21913185 10.1002/ijc.26422

[28] Matsuzaki J, Kato K, Oono K, Tsuchiya N, Sudo K, Shimomura A, et al. Prediction of tissue-of-origin of early stage cancers using serum miRNomes. JNCI Cancer Spectr. 2023;7. 10.1093/jncics/pkac080. 10.1093/jncics/pkac080 PMC9825310 36426871

[29] Schultz NA, Dehlendorff C, Jensen BV, Bjerregaard JK, Nielsen KR, Bojesen SE, et al. MicroRNA biomarkers in whole blood for detection of pancreatic cancer. JAMA. 2014;311:392–404. 24449318 10.1001/jama.2013.284664

[30] Nakamura K, Zhu Z, Roy S, Jun E, Han H, Munoz RM, et al. An exosome-based transcriptomic signature for noninvasive, early detection of patients with pancreatic ductal adenocarcinoma: a multicenter cohort study. Gastroenterology. 2022;163:1252–1266.e2. 35850192 10.1053/j.gastro.2022.06.090 PMC9613527

[31] Suzuki K, Igata H, Abe M, Yamamoto Y, Iwanaga T, Kanzaki H, et al. Multiple cancer type classification by small RNA expression profiles with plasma samples from multiple facilities. Cancer Sci. 2022;113:2144–66. 35218669 10.1111/cas.15309 PMC9207371

[32] Tsuzuki S, Fujitsuka N, Horiuchi K, Ijichi S, Gu Y, Fujitomo Y, et al. Factors associated with sufficient knowledge of antibiotics and antimicrobial resistance in the Japanese general population. Sci Rep. 2020;10. 10.1038/s41598-020-60444-1. 10.1038/s41598-020-60444-1 PMC7044168 32103110

[33] Muhlestein WE, Akagi DS, Davies JM, Chambless LB. Predicting inpatient length of stay after brain tumor surgery: developing machine learning ensembles to improve predictive performance. Clin Neurosurg. 2019;85:384–93. 10.1093/neuros/nyy343 PMC7137462 30113665

[34] Zou H, Hastie T. Regularization and variable selection via the elastic net. J. R. Statist. Soc. B. 2005:67;301–20.

[35] Deng L, Pan J, Xu X, Yang W, Liu C, Liu H. PDRLGB: Precise DNA-binding residue prediction using a light gradient boosting machine. BMC Bioinform. 2018;19. 10.1186/s12859-018-2527-1. 10.1186/s12859-018-2527-1 PMC6311926 30598073

[36] Chen K, Li R, Dou Y, Liang Z, Lv Q. Ranking support vector machine with kernel approximation. Comput Intell Neurosci. 2017;2017. 10.1155/2017/4629534. 10.1155/2017/4629534 PMC5331172 28293256

[37] Marée R, Geurts P, Wehenkel L. Random subwindows and extremely randomized trees for image classification in cell biology. BMC Cell Biol. 2007;8. 10.1186/1471-2121-8-S1-S2. 10.1186/1471-2121-8-S1-S2 PMC1924507 17634092

[38] Zagoruyko S, Komodakis N. Wide Residual Networks. 2016 http://arxiv.org/abs/1605.07146.

[39] Li W, Liu H, Yang P, Xie W. Supporting regularized logistic regression privately and efficiently. PLoS ONE. 2016;11. 10.1371/journal.pone.0156479. 10.1371/journal.pone.0156479 PMC4894560 27271738

[40] Albaradei S, Thafar M, Alsaedi A, Van Neste C, Gojobori T, Essack M, et al. Machine learning and deep learning methods that use omics data for metastasis prediction. Comput Struct Biotechnol J. 2021;19:5008–18. 34589181 10.1016/j.csbj.2021.09.001 PMC8450182

[41] Chen T, Guestrin C. XGBoost: A Scalable Tree Boosting System. 2016. 10.1145/2939672.2939785.

[42] Friedman JH, Popescu BE. Predictive learning via rule ensembles. Ann Appl Stat. 2008;2:916–54.

[43] Krauss C, Do XA, Huck N. Deep neural networks, gradient-boosted trees, random forests: Statistical arbitrage on the S&P 500. Eur J Oper Res. 2017;259:689–702.

[44] Sachdeva S, Kumar B. A comparative study between frequency ratio model and gradient boosted decision trees with greedy dimensionality reduction in groundwater potential assessment. Water Resour Manag. 2020;34:4593–615.

[45] Breiman L. Random Forests. Machine Learning 2001;45:5–32.

[46] Bloomston M, Frankel WL, Petrocca F, Volinia S, Alder H, Hagan JP, et al. MicroRNA expression patterns to differentiate pancreatic adenocarcinoma from normal pancreas and chronic pancreatitis. https://jamanetwork.com/. 10.1001/jama.297.17.1901 17473300

[47] Androvic P, Benesova S, Rohlova E, Kubista M, Valihrach L. Small RNA-sequencing for analysis of circulating miRNAs. J Mol Diagn. 2022;24:386–94. 35081459 10.1016/j.jmoldx.2021.12.006

[48] Wright C, Rajpurohit A, Burke EE, Williams C, Collado-Torres L, Kimos M, et al. Comprehensive assessment of multiple biases in small RNA sequencing reveals significant differences in the performance of widely used methods. BMC Genom. 2019;20:513. 10.1186/s12864-019-5870-3 PMC6588940 31226924

[49] Mestdagh P, Hartmann N, Baeriswyl L, Andreasen D, Bernard N, Chen C, et al. Evaluation of quantitative miRNA expression platforms in the microRNA quality control (miRQC) study. Nat Methods. 2014;11:809–15. 24973947 10.1038/nmeth.3014

