# Supplementary material for: Early detection of pancreatic cancer by comprehensive serum miRNA sequencing with automated machine learning
Source: Br J Cancer. 2024 Aug 28;131(7):1158–68. doi: 10.1038/s41416-024-02794-5 (PMC11442445; doi:10.1038/s41416-024-02794-5)
Supplement: Supplementary file 3 — supplemental Table 3 [file 41416_2024_2794_MOESM3_ESM.docx]

| **Supplementary Table 3. Statistical analysis for the characteristics of the training cohort vs. validation cohort in pancreatic cancers and healthy controls, respectively.** | | | |
| --- | --- | --- | --- |
| **P-value (training vs validation)** | **PC** | **HC** |  |
| Sex | .981^b^ | .244^b^ |  |
| Age | .596^a^ | .645^a^ |  |
| History of smoking | .539^b^ | .552^b^ |  |
| Drinking habits | .086^b^ | .267^b^ |  |
| Diabetes mellitus | .609^b^ | .972^b^ |  |
| CA19-9 | .311^a^ | .209^a^ |  |
| CEA | .243^a^ | .795^a^ |  |
| DUPAN-2 | .323^a^ | .852^a^ |  |
| Stage | .016^b^ | - |  |
| PC; Pancreatic Cancer, HC; Healthy Control. ^a^ Student’s t-test. ^b^ Pearson’s chi-squared test. | | |  |
