# Supplementary material for: Early detection of pancreatic cancer by comprehensive serum miRNA sequencing with automated machine learning
Source: Br J Cancer. 2024 Aug 28;131(7):1158–68. doi: 10.1038/s41416-024-02794-5 (PMC11442445; doi:10.1038/s41416-024-02794-5)
Supplement: Supplementary file 4 — supplemental Table 4 [file 41416_2024_2794_MOESM4_ESM.docx]

| **Supplementary Table 4. The performance of serum CA19-9, miRNA model, and miRNA+CA19-9 model in the diagnosis of early-stage pancreatic cancers from healthy participants.** | | | |
| --- | --- | --- | --- |
|  | **CA19-9** | **miRNA model** | **miRNA+CA19-9 model** |
| Specificity | 0.98^a^ | 0.98^b^ | 0.98^b^ |
| 95% CI | 0.96-1.00 | - | - |
| AUC |  |  |  |
| Stage 0-I | 0.81 | 0.92 | 0.98 |
| 95% CI | 0.71-0.92 | 0.86-0.98 | 0.96-1.00 |
| P-value | ref | .184 | .001 |
| Stage 0-II | 0.84 | 0.92 | 0.98 |
| 95% CI | 0.76-0.91 | 0.87-0.96 | 0.97-1.00 |
| P-value | ref | .207 | < .001 |
| Sensitivity |  |  |  |
| Stage 0-I | 0.23 | 0.60 | 0.77 |
| 95% CI | 0.10-0.40 | 0.43-0.80 | 0.60-0.90 |
| P-value | ref | .020 | < .001 |
| Stage 0-II | 0.45 | 0.50 | 0.85 |
| 95% CI | 0.33-0.57 | 0.37-0.75 | 0.73-0.93 |
| P-value | ref | 1 | < .001 |
| ^a^ Threshold = 37 U/mL. ^b^ The same specificity as that of CA19-9. | | | |
