# Supplementary material for: Early detection of pancreatic cancer by comprehensive serum miRNA sequencing with automated machine learning
Source: Br J Cancer. 2024 Aug 28;131(7):1158–68. doi: 10.1038/s41416-024-02794-5 (PMC11442445; doi:10.1038/s41416-024-02794-5)
Supplement: Supplementary file 5 — supplemental Table 5 [file 41416_2024_2794_MOESM5_ESM.docx]

| **Supplementary Table 5. The performance of serum CA19-9, miRNA model, and miRNA+CA19-9 model to discriminate asymptomatic pancreatic cancers in stage 0-I from healthy participants.** | | | |
| --- | --- | --- | --- |
| **Asymptomatic, stage 0-I PC** | **CA19-9** | **miRNA model** | **miRNA+CA19-9 model** |
| Specificity | 0.98^a^ | 0.98^b^ | 0.98^b^ |
| 95% CI | 0.96-1.00 | - | - |
| AUC | 0.81 | 0.89 | 0.97 |
| 95% CI | 0.70-0.93 | 0.81-0.97 | 0.95-1.00 |
| P-value | ref | .659 | .007 |
| Sensitivity | 0.29 | 0.48 | 0.67 |
| 95% CI | 0.10-0.57 | 0.29-0.71 | 0.48-0.86 |
| P-value | ref | .578 | .027 |
| PC; Pancreatic Cancer. ^a^ Threshold = 37 U/mL. ^b^ The same specificity as that of CA19-9. | | | |
