# Supplementary material for: Early detection of pancreatic cancer by comprehensive serum miRNA sequencing with automated machine learning
Source: Br J Cancer. 2024 Aug 28;131(7):1158–68. doi: 10.1038/s41416-024-02794-5 (PMC11442445; doi:10.1038/s41416-024-02794-5)
Supplement: Supplementary file 6 — supplemental Table 6 [file 41416_2024_2794_MOESM6_ESM.docx]

**Supplementary Table 6. The performance of miRNA model and miRNA+CA19-9 model to discriminate pancreatic cancer patients from healthy participants whose miRNA expression was measured by the Thermo Fisher NGS platform.**

|  | **miRNA model** | | | | **miRNA+CA19-9 model** | | | |
| --- | --- | --- | --- | --- | --- | --- | --- | --- |
| AUC | 0.93 | | | | 0.98 | | | |
| 95% CI | 0.90-0.96 | | | | 0.96-0.99 | | | |
| Specificity | 0.85 | 0.90 | 0.95 | 0.98 | 0.85 | 0.90 | 0.95 | 0.98 |
| All stages sensitivity | 0.83 | 0.78 | 0.68 | 0.63 | 0.95 | 0.91 | 0.85 | 0.82 |
| 95% CI | 0.73-0.92 | 0.68-0.88 | 0.56-0.82 | 0.48-0.74 | 0.88-0.99 | 0.85-0.98 | 0.78-0.95 | 0.75-0.91 |
| Each stage sensitivity |  |  |  |  |  |  |  |  |
| Stage 0 | 0.67 | 0.50 | 0.50 | 0.50 | 0.67 | 0.50 | 0.50 | 0.50 |
| 95% CI | 0.33-1.00 | 0.17-1.00 | 0.17-0.83 | 0.17-0.83 | 0.33-1.00 | 0.17-1.00 | 0.17-0.83 | 0.17-0.83 |
| Stage I | 0.88 | 0.79 | 0.63 | 0.58 | 0.96 | 0.92 | 0.79 | 0.75 |
| 95% CI | 0.71-1.00 | 0.58-0.96 | 0.42-0.88 | 0.33-0.79 | 0.83-1.00 | 0.79-1.00 | 0.63-1.00 | 0.58-0.92 |
| Stage II | 0.70 | 0.67 | 0.60 | 0.47 | 0.97 | 0.90 | 0.83 | 0.77 |
| 95% CI | 0.53-0.87 | 0.50-0.83 | 0.37-0.80 | 0.27-0.70 | 0.83-1.00 | 0.80-1.00 | 0.70-0.97 | 0.60-0.93 |
| Stage III | 0.93 | 0.87 | 0.77 | 0.70 | 0.97 | 0.97 | 0.93 | 0.90 |
| 95% CI | 0.80-1.00 | 0.70-1.00 | 0.57-0.93 | 0.43-0.87 | 0.90-1.00 | 0.90-1.00 | 0.83-1.00 | 0.80-1.00 |
| Stage IV | 0.87 | 0.83 | 0.77 | 0.77 | 0.97 | 0.93 | 0.90 | 0.90 |
| 95% CI | 0.70-0.97 | 0.67-0.97 | 0.60-0.93 | 0.57-0.90 | 0.87-1.00 | 0.83-1.00 | 0.80-1.00 | 0.80-1.00 |
