# Supplementary material for: Early detection of pancreatic cancer by comprehensive serum miRNA sequencing with automated machine learning
Source: Br J Cancer. 2024 Aug 28;131(7):1158–68. doi: 10.1038/s41416-024-02794-5 (PMC11442445; doi:10.1038/s41416-024-02794-5)
Supplement: Supplementary file 7 — supplemental Table 7 [file 41416_2024_2794_MOESM7_ESM.docx]

**Supplementary Table7. The performance of miRNA model and miRNA+CA19-9 model constructed by data from the Thermo Fisher NGS platform to discriminate pancreatic cancer patients from healthy participants.**

|  | **miRNA model** | | | | **miRNA+CA19-9 model** | | | | |
| --- | --- | --- | --- | --- | --- | --- | --- | --- | --- |
| AUC | 0.93 | | | | 0.98 | | | | |
| 95% CI | 0.91-0.96 | | | | 0.96-0.99 | | | | |
| Specificity | 0.85 | 0.90 | 0.95 | 0.98 | 0.85 | 0.90 | 0.95 | 0.98 |  |
| All stages sensitivity | 0.86 | 0.78 | 0.72 | 0.63 | 0.94 | 0.93 | 0.87 | 0.85 |  |
| 95% CI | 0.74-0.93 | 0.70-0.89 | 0.60-0.83 | 0.54-0.78 | 0.89-0.98 | 0.84-0.98 | 0.80-0.95 | 0.77-0.92 |  |
| Each stage sensitivity |  |  |  |  |  |  |  |  |  |
| Stage 0 | 0.50 | 0.50 | 0.50 | 0.50 | 0.83 | 0.83 | 0.50 | 0.50 |  |
| 95% CI | 0.17-0.83 | 0.17-0.83 | 0.17-0.83 | 0.17-0.83 | 0.50-1.00 | 0.33-1.00 | 0.17-1.00 | 0.17-0.83 |  |
| Stage I | 0.88 | 0.83 | 0.75 | 0.58 | 0.96 | 0.92 | 0.79 | 0.79 |  |
| 95% CI | 0.71-1.00 | 0.67-0.96 | 0.50-0.96 | 0.33-0.88 | 0.79-1.00 | 0.75-1.00 | 0.63-0.96 | 0.58-0.96 |  |
| Stage II | 0.77 | 0.60 | 0.60 | 0.50 | 0.93 | 0.93 | 0.83 | 0.77 |  |
| 95% CI | 0.50-0.93 | 0.43-0.87 | 0.37-0.77 | 0.33-0.70 | 0.87-1.00 | 0.77-1.00 | 0.70-0.97 | 0.63-0.93 |  |
| Stage III | 0.93 | 0.90 | 0.77 | 0.73 | 0.97 | 0.97 | 0.97 | 0.97 |  |
| 95% CI | 0.83-1.00 | 0.77-1.00 | 0.60-0.97 | 0.57-0.90 | 0.90-1.00 | 0.90-1.00 | 0.90-1.00 | 0.80-1.00 |  |
| Stage IV | 0.93 | 0.83 | 0.80 | 0.73 | 0.93 | 0.93 | 0.93 | 0.93 |  |
| 95% CI | 0.77-1.00 | 0.70-0.97 | 0.63-0.93 | 0.60-0.90 | 0.83-1.00 | 0.83-1.00 | 0.83-1.00 | 0.83-1.00 |  |
