# Supplementary material for: Early detection of pancreatic cancer by comprehensive serum miRNA sequencing with automated machine learning
Source: Br J Cancer. 2024 Aug 28;131(7):1158–68. doi: 10.1038/s41416-024-02794-5 (PMC11442445; doi:10.1038/s41416-024-02794-5)
Supplement: Supplementary file 8 — supplemental Figures [file 41416_2024_2794_MOESM8_ESM.pdf]

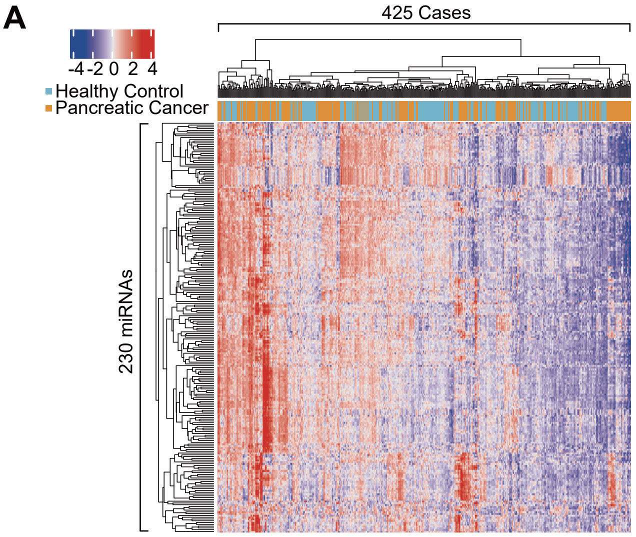

Supplementary Figure 1.

The serum miRNA expression profiles in healthy controls differed from those in pancreatic cancer patients. (A) Heatmap representing hierarchical unsupervised clustering analysis of the miRNA sequencing of healthy controls (blue) and pancreatic cancer patients (orange).

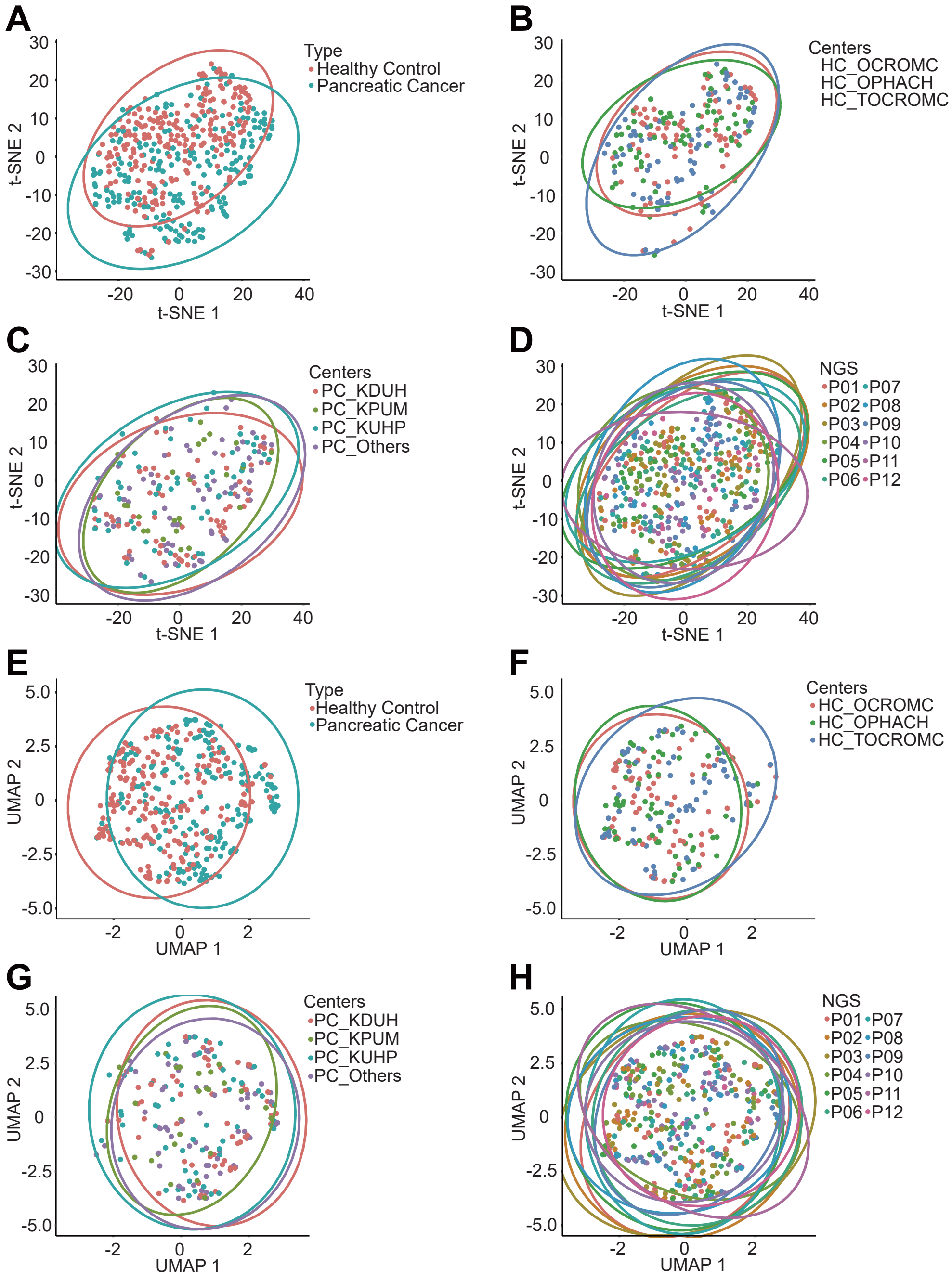

Supplementary Figure 2.

The miRNA expression profiles were distinct between healthy controls and pancreatic cancer patients but not significantly different in the blood collection centers and NGS measurements. (A) T-distributed stochastic neighbor embeddings (t-SNEs) of the miRNA sequencing of healthy controls (blue) and pancreatic cancer patients (orange). (B-D) The t-SNEs grouped by clinics, hospitals, and the NGS measurements. HC; healthy control, PC; pancreatic cancer. (E) Uniform manifold approximation and projection (UMAP) of the miRNA sequencing of healthy controls (blue) and pancreatic cancer patients (orange). (F-H) The UMAP grouped by clinics, hospitals, and the NGS measurements. HC; healthy control, PC; pancreatic cancer.

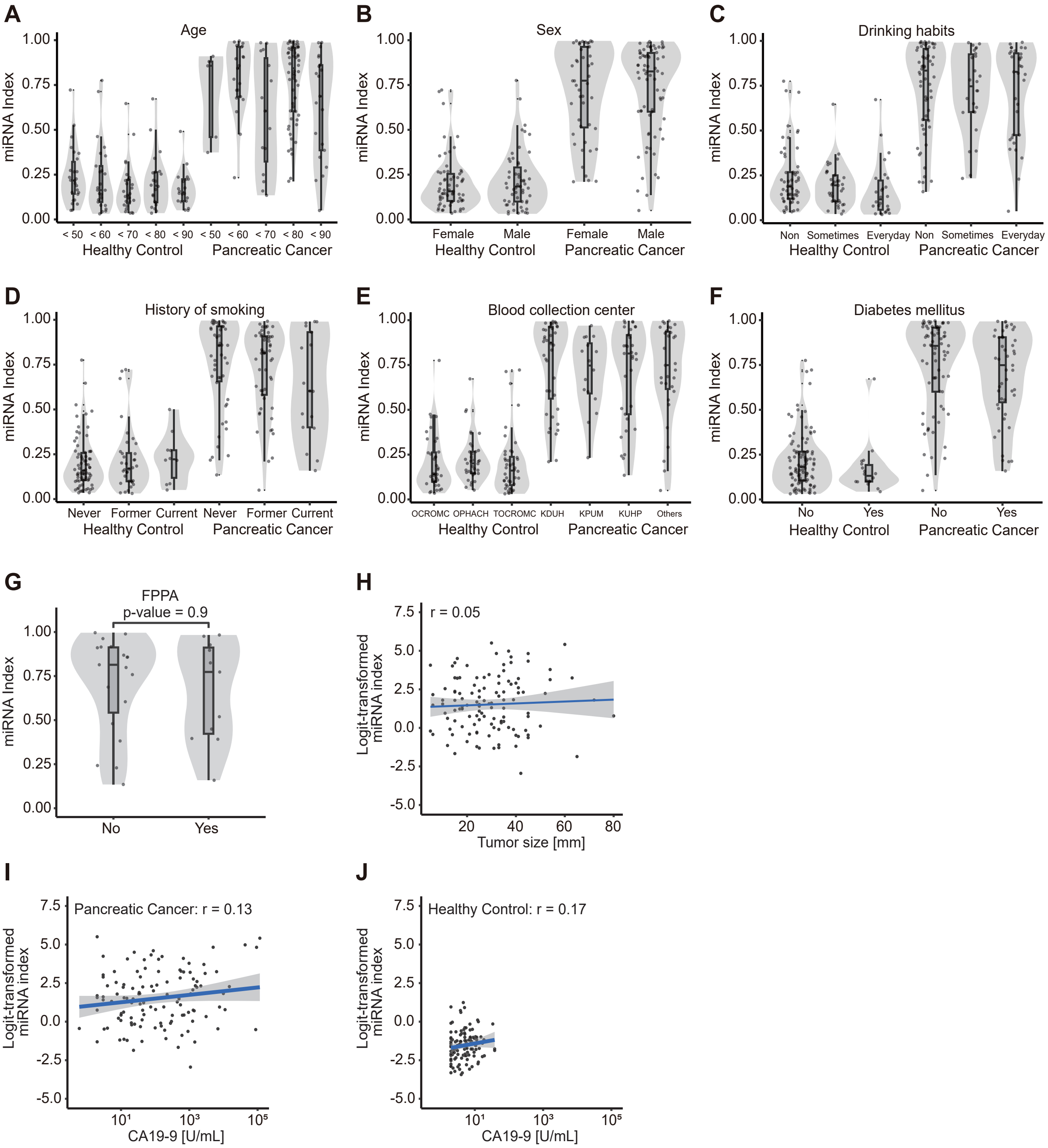

Supplementary Figure 3.

No association between indices of the miRNA model and age, sex, drinking habits, history of smoking, blood collection center, diabetes mellitus status, focal pancreatic parenchymal atrophy (FPPA), tumor size, or CA19-9 level. (A-G) Box plots of indices of the miRNA model of healthy participants and pancreatic cancers in the validation cohort, separately drawn based on patient backgrounds of (A) age, (B) sex, (C) drinking habits, (D) history of smoking, (E) blood collection centers, (F) diabetes mellitus, and (G) FPPA. (H-J) Scatter plots of indices of the miRNA model and (H) tumor size of pancreatic cancers, (I) CA19-9 levels of pancreatic cancer patients, and (J) CA19-9 levels of healthy participants in the validation cohort, drawn with linear regression line and 95% confidence interval.

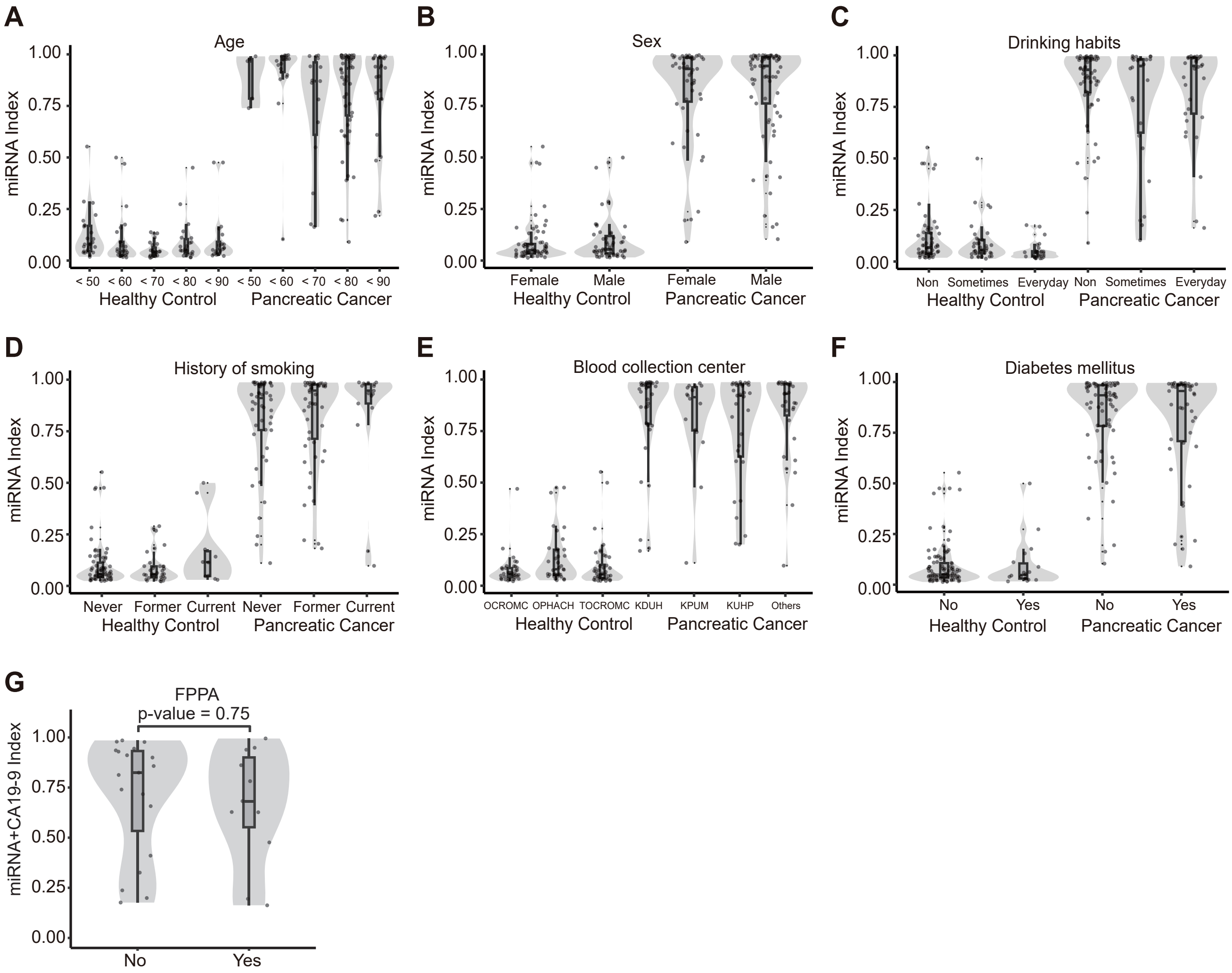

Supplementary Figure 4.

No association between indices of the miRNA+CA19-9 model and age, sex, drinking habits, history of smoking, blood collection center, diabetes mellitus status, focal pancreatic parenchymal atrophy (FPPA). (A-G) Box plots of indices of the miRNA+CA19-9 model of healthy participants and pancreatic cancer patients in the validation cohort, separately drawn based on patient backgrounds of (A) age, (B) sex, (C) drinking habits, (D) history of smoking, (E) blood collection centers, (F) diabetes mellitus, and (G) FPPA.

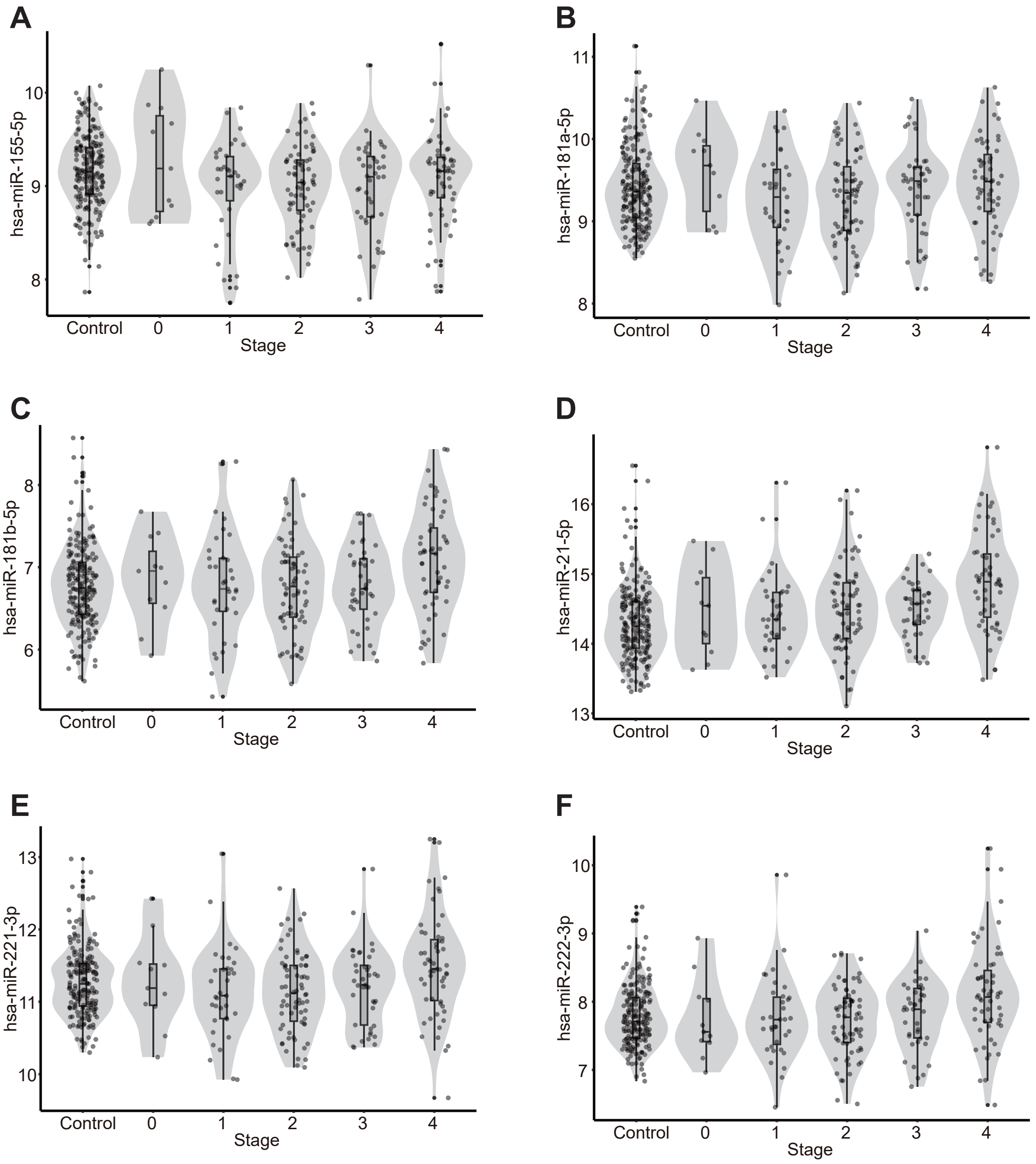

Supplementary Figure 5.

Some of the miRNAs upregulated in the tumor were also upregulated in the serum. (A-F) Box plots of the expression of hsa-miR-155-5p (A), hsa-miR-181a-5p (B), hsa-miR-181b-5p (C), hsa-miR-21-5p (D), hsa-miR-221-3p (E), and hsa-miR-222-3p (F) in the serum of healthy controls and pancreatic cancer patients with each stage.

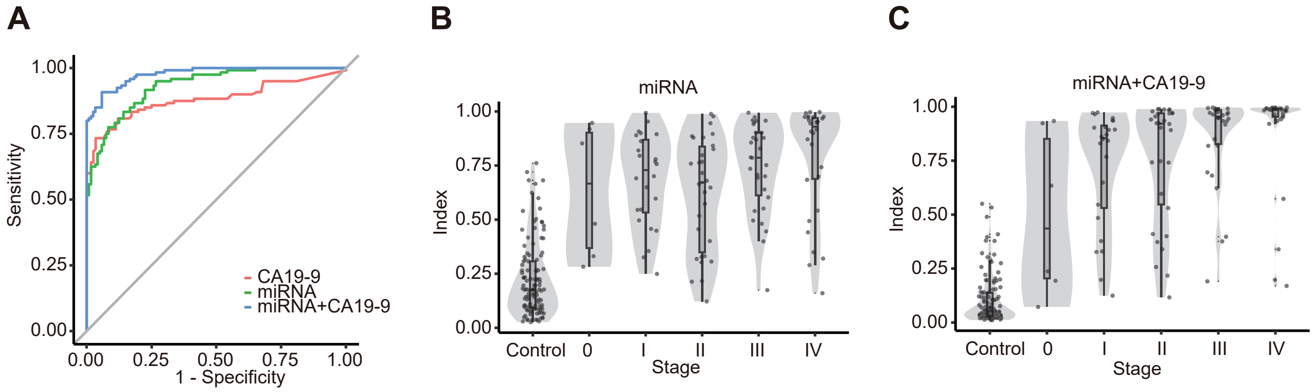

Supplementary Figure 6.

The miRNA and miRNA+CA19-9 models demonstrated high performance on the validation cohort measured with the Thermo Fisher NGS platform. (A) ROC curve for the serum CA19-9 alone (red), the miRNA model (green), and the miRNA+CA19-9 model (blue) in the validation cohort measured with the Thermo Fisher NGS platform (Ion GeneStudio S5 system). (B-C) Box plots of indices of the miRNA model and the miRNA+CA19-9 model of healthy participants and pancreatic cancer patients in each stage (0, I, II, III, and IV) in the validation cohort measured with the Thermo Fisher NGS platform (Ion GeneStudio S5 system).

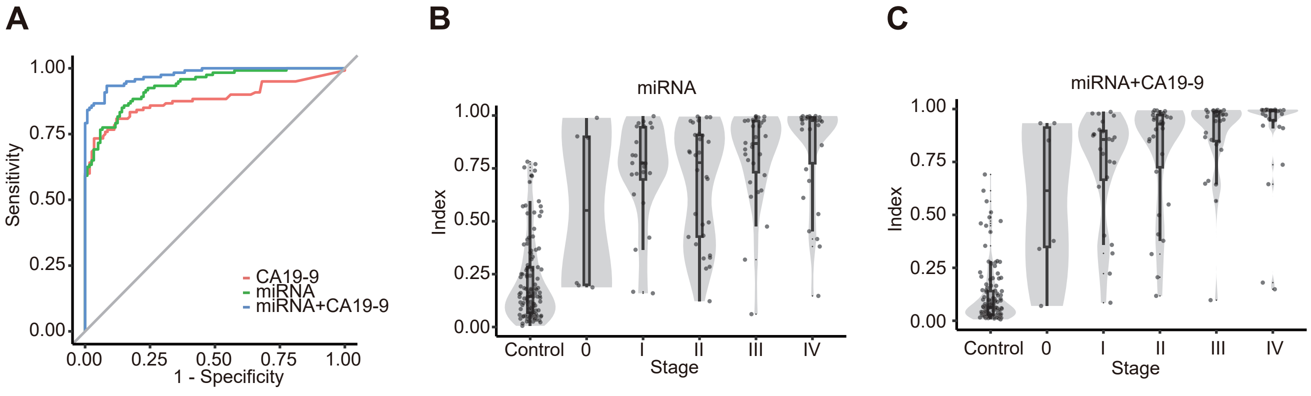

Supplementary Figure 7.

New models we created using miRNA expressions measured with the Thermo Fisher NGS platform demonstrated high performance comparable to those constructed by the NextSeq 550 data. (A) ROC curves for the serum CA19-9 alone (red), the new miRNA model (green), and the new miRNA+CA19-9 model (blue) in the validation cohort measured with the Thermo Fisher NGS platform (Ion GeneStudio S5 system). (B-C) Box plots of indices of the new miRNA and miRNA+CA19-9 models of healthy participants and pancreatic cancer patients in each stage (0, I, II, III, and IV) in the validation cohort measured with the Thermo Fisher NGS platform (Ion GeneStudio S5 system).
